# Radiative and non-radiative transitions of excited Ti^3+^ cations in sapphire

**DOI:** 10.1038/s41598-019-55267-8

**Published:** 2019-12-11

**Authors:** Avry Shirakov, Zeev Burshtein, Yehoshua Shimony, Eugene Frumker, Amiel A. Ishaaya

**Affiliations:** 1 Department of Physics, Ben-Gurion University of the Negev, Beer-Sheva, 84105 Israel; 2 Department of Electrical and Computer Engineering, Ben-Gurion University of the Negev, Beer-Sheva, 84105 Israel; 3 Department of Materials Engineering, Ben-Gurion University of the Negev, Beer-Sheva, 84105 Israel; 4 Applied Physics Division, Soreq NRC, Yavne, 81800 Israel

**Keywords:** Solid-state lasers, Atomic and molecular interactions with photons

## Abstract

We have measured the fluorescence quantum efficiency in Ti^3+^:sapphire single crystals between 150 K and 550 K. Using literature-given effective fluorescence lifetime temperature dependence, we show that the zero temperature radiative lifetime is (4.44 ± 0.04) *μs*, compared to the 3.85 *μs* of the fluorescence lifetime. Fluorescence lifetime thermal shortening resolves into two parallel effects: radiative lifetime shortening, and non-radiative transition rate enhancement. The first is due to thermally enhanced occupation of a Δ*E* = 1,700 cm^−1^ higher (top) electronic state of the upper multiplet, exhibiting a transition oscillator strength of *f* = 0.62, compared to only 0.013 of the bottom electronic state of the same multiplet. The non-radiative rate relates to multi-phonon decay transitions stimulated by the thermal phonon occupation. Thermal enhancement of the configuration potential anharmonicity is also observed. An empiric expression for the figure-of-anharmonicity temperature dependence is given as $\hat{{\bf{H}}}$ (*T*) = $\hat{{\bf{H}}}$ (0)(1 + *β* exp(−ℏ*ω*_*co*_* /k*_*B*_*T* )), where $\hat{{\bf{H}}}$ (0) = 0.276, *β* = 5.2, ℏ*ω*_*co*_ = 908 cm^−1^, and *k*_*B*_ is the Boltzmann constant.

## Introduction

The spectroscopic properties of Ti:sapphire crystals have been thoroughly studied, partially due to its broad lasing tunability^1–4^, and capability to produce ultra-short laser pulses in the femto-second (fs) range^5–8^. The crystal exhibits a broad (FWHM) absorption band between about 450 nm and 570 nm, and a broad fluorescence emission band between about 680 nm and 850 nm.

The fluorescence decay time (effective lifetime) versus temperature dependence has been measured by quite a number of other workers^9–12^. The decay time was quite constant (3.85 *μ*s) between the absolute zero temperature and about 200 K, then started to shorten, becoming approximately 44 ns at 633 K. The near absolute zero fluorescence lifetime has been favorably interpreted to represent the pure radiative lifetime of the optical transition from the excited upper multiplet electronic states down to the ground states multiplet. The lifetime shortening has been attributed to thermally activated non-radiative transitions competing with the radiative one^10,11^.

Measurements of heat generation by conversion of excited states energy into thermal energy at room temperature (T = 300 K) provided a fluorescence quantum efficiency estimate of approximately 69.5%^13^, or 68% at 325 K^9^. Using the measured cavity losses of a continuous wave (CW) Ti:sapphire laser, the fluorescence quantum efficiency was estimated as (64 ± 10)%^2^. The similarity between the above estimates and the effective (measured) fluorescence lifetime ratios *τ*_*eff*_(*T*)/*τ*_*eff*_(0) was considered close enough to support both the identification of *τ*_*eff*_(0) as a pure radiative lifetime *τ*_*rad*_, and the lifetime shortening as due to non-radiative transitions.

Various theoretical analyses undertook empiric or quasi-empiric approaches. Byvik *et al*.^10^ described the radiative lifetime *τ*_*eff*_ as

1 $${\tau }_{eff}^{-1}={\tau }_{rad}^{-1}+\alpha \cdot \exp (-\delta E/{k}_{B}T).$$

The second addend describes the thermally activated non-radiative transition by a two-level system. The experimental results provide a best fit using *δE* = 1,300 cm^−1^, yet no specific physical assignment is provided for that particular energy value. Albers *et al*.^9^ described the non-radiative process as initiated by tunneling from the upper multiplet electronic state configuration potential to the lower one at a higher vibrational state. Then, the higher vibrational state thermalizes to the lowest one by emitting its excess energy to the host matrix vibrations. The final expression is of the form

2 $${\tau }_{eff}^{-1}={\tau }_{rad}^{-1}+{\tau }_{n{r}_{0}}^{-1}{W}_{p}(\langle m\rangle ,S),$$

where ${\tau }_{n{r}_{0}}^{-1}$ is a non-radiative rate factor, *W*_*p*_ is a Huang-Rhys-Pekar (HRP) function^14^, 〈*m*〉 is Planck’s average thermal occupancy of the vibrational states, and *S* is the Huang-Rhys parameter, which measures the Franck-Condon offset between initial and final states.

In the present work we have measured the fluorescence quantum efficiency over a considerably broad region of temperatures (150–550 K). We have found that the change in the effective (measured) fluorescence lifetime *τ*_*eff*_ is caused by mixed changes in the radiative lifetime *τ*_*rad*_ and in the non-radiative lifetime *τ*_*nr*_. The latter is interpreted on the basis of a theory that considers the excited states interactions with the host crystalline material phonon field as a source for a multi phonon non-radiative decay^15^. That particular theory was found to be quite satisfactory in accounting for reported transitions, involving energy gaps of up to approximately 5,000 cm^−1^. In a recent publication, it was successfully applied to analysis of fluorescence lifetime temperature dependence of Co^2+^ excited states in an AgCl_0.5_Br_0.5_ matrix^16^, involving an energy gap of about 2,800 cm^−1^. Obviously, energy gaps of Ti:sapphire, approximately 11,500 cm^−1^, are considerably larger.

## Experimental Details

The Ti:sapphire used in the present study was of the form of a 4 × 4 × 10 mm^3^ crystalline rod with Brewster-cut edges and all surfaces optically polished. This particular sample previously served as the amplifying medium in a lab-designed tunable laser^17,18^.

**Figure 1:**
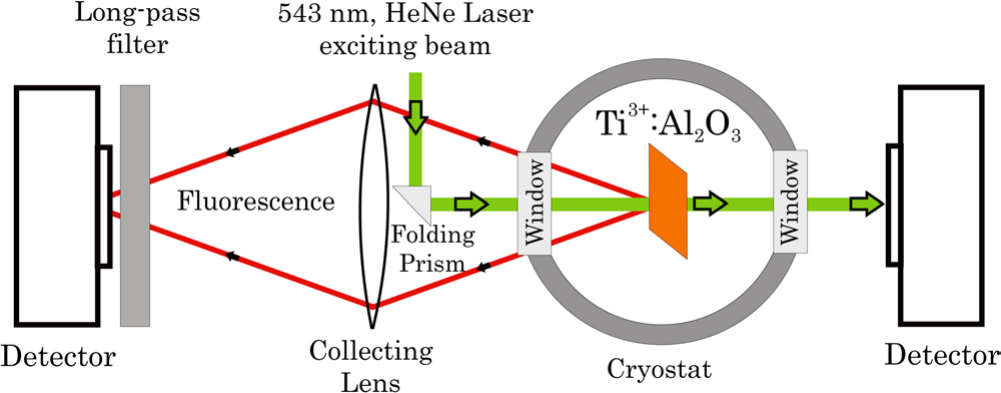
Schematic layout of the Ti:sapphire quantum efficiency measurement setup; for details see text.

Figure 1 provides a layout of the low-temperature measuring system. The fluorescence exciting CW beam was of 543 nm wavelength from a Laser Research model 2 mW He-Ne laser. The powers of the input and the sample-crossing 543 nm beams were measured using a Thorlabs PDA100A photodiode. Cooling was obtained under vacuum by using a lab-designed liquid nitrogen cryostat between room temperature and about 150 K. The collected fluorescence power was measured by an Ophir PD300-SH photodiode laser-measurement sensor. The fluorescence emission was collected using a BK7 lens of 50 mm diameter and 50 mm focal length. Scattered and ambient light traces were filtered out using a Schott Glaswerke 650 nm long-pass filter. The above room temperature measurements system configuration was quite similar. Sample heating up to 550 K was performed in free air using a standard heating plate. Scattered and ambient light traces were filtered out using a Corning No. 3–67 long-pass filter (*λ* > 560 nm).

The sample temperature was measured using two T-type thermocouples: one attached directly to the sample (pressed against the sample), several millimeter away from the crossing fluorescence excitation beam; the other was attached to the copper-made holder; notably, both copper and sapphire exhibit quite a large thermal conductivity. The resultant error was better than about ±1 K.

The total fluorescence power was estimated by correcting against the inner Ti:sapphire Fresnel reflectances, the collecting lens solid angle collection efficiency, and other reflection and absorption loss factors of the system. Light polarization relations have been overlooked. The fluorescence quantum efficiency is derived from the ratio between the estimated total fluorescence photon emission rate and the absorption rate of exciting photons. The room temperature fluorescence quantum efficiency is nominally estimated as (50 ± 10)%. The temperature dependence of the fluorescence power shape is, however, much more accurate. To obtain a fair estimate of the quantum efficiency throughout the entire temperature range, we have normalized all thus-acquired values to the room temperature quantum efficiency value ((69.5 ± 1)%) measured by Y. Li *et al*.^13^ using a photo-calorimetric compensation technique. That method measures directly the material heating due to the non-radiative excited states decay transitions, and is thus bound to provide a most reliable estimate of the non-radiative losses. Unfortunately, measurements using that particular method have been performed only at the room temperature vicinity.

## Ti:Sapphire Energy Scheme Presentation

In the following we provide a somewhat revised energy scheme interpretation of the Ti^3+^ cations based on the room temperature measured spectra by Moulton^11^. The host sapphire (Al_2_O_3_) crystal belongs to the rhombohedral #167D_3d_^6^ (*R*$\bar{3}$2/*c*) space-group. The Al^3+^ cations occupy sites of C_3_ symmetry, octahedrally coordinated by 6 nearest-neighbors O^2−^ anions^19^. The Ti^3+^ cations replace the lattice aluminum Al^3+^ in its site. The free-ion valence electronic state Term is ^2^D. The resulting crystal field splitting is usually considered through a succession of perturbations, as described below.

**Figure 2:**
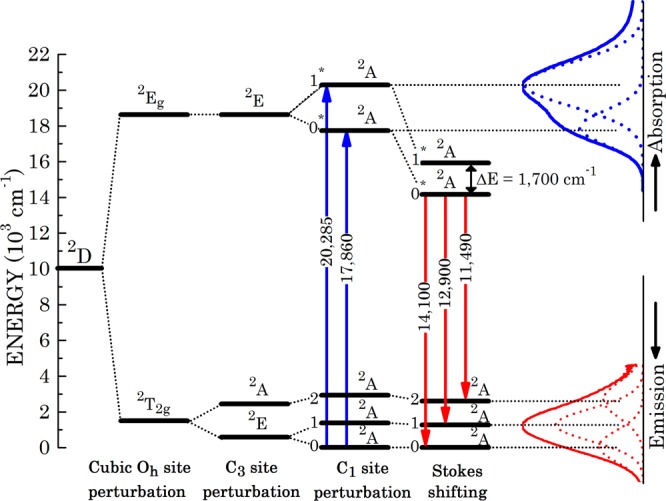
Energy state diagram of the Ti^3+^ ions in the sapphire matrix. The state energies scheme stems from analysis of the raw data taken from the original paper by Moulton^11^. The ground multiplet levels are tagged 0, 1, 2, at an increasing energy order. The upper multiplet levels are correspondingly tagged 0^*^, 1^*^, replotted and resolved on the right side of the figure. The 0^*^–1^*^ energy separation Δ*E* post Stokes shifting, which is a result of analysis performed in our present paper, has also been included.

A zero-order perturbation for the crystal field splitting takes advantage of the fact that the site is octahedrally coordinated. It is correspondingly assumed to exhibit an octahedral O_h_ symmetry; the state splitting is ^2^D → ^2^E_g_ ⊕ ^2^T_2g_. In a further order perturbation, the site symmetry is assumed to be identical with that of the native Al^3+^, namely a C_3_ symmetry. This converts the ^2^E_g_ state according to ^2^E_g_ → ^2^E, and splits the ^2^T_2g_ state according to ^2^T_2g_ → ^2^E ⊕ ^2^A. The titanium Ti^3+^ ionic radius is however much greater than the native aluminum ion one; 0.67 Å and 0.535 Å, respectively^20^. That gross, about 25% difference, induces a further site-symmetry reduction, namely into a C_1_ symmetry. In that respect, a Hume-Rothery empiric criterion rule was administered^21^, namely that a site accepting a dopant ion may preserve its symmetry only if the difference between the dopant and native ions radiuses never exceeds about 15%. The upper ^2^E state splits in two, according to ^2^E → 2^2^A. The lower ^2^T_2g_ state eventually transforms into a 3^2^A multiplet (ground multiplet), and the upper ^2^E_g_ state eventually transforms into a 2^2^A multiplet (upper multiplet). All optical transitions among the states are symmetry allowed. All states are strongly broadened by interaction with lattice vibrations, as indicated in Fig. 2. The “0−0^*^” optical inter-multiplet transitions exhibit a strong Stokes shift from 17,857 cm^−1^ down to 14,100 cm^−1^. That strong shift is an additional persuading indication of the strong electronic states interaction with the lattice vibrations. This is also the reason why no spin-orbit splitting of any of the ^2^A dublet states could be spectroscopically resolved by neither absorption nor fluorescence emission spectra.

Notably, quite a number of authors assigned the ^2^E splitting to a Jahn-Teller effect^11,22,23^. Our present assignment to a site distortion by the radiuses mismatch between Ti^3+^ and the native Al^3+^ is much more reasonable, obviating the need to invoke a Jahn-Teller splitting effect. It should be practically wise to adopt a comment made by Van Vleck^24^ that “It is great merit of the Jahn-Teller effect that it disappears when not needed”. Sorting out of this problem does not belong to the subject matter of our present work. Nevertheless, it would be interesting to theoretically assess the contribution of the Jahn-Teller effect in the specific case of Ti^3+^ in sapphire. It may also be quite interesting to assess theoretically the distorted structure of the originally C_3_ symmetry site of the Al^3+^ in sapphire upon replacement by a Ti^3+^ dopant.

## Results and Discussion

**Figure 3:**
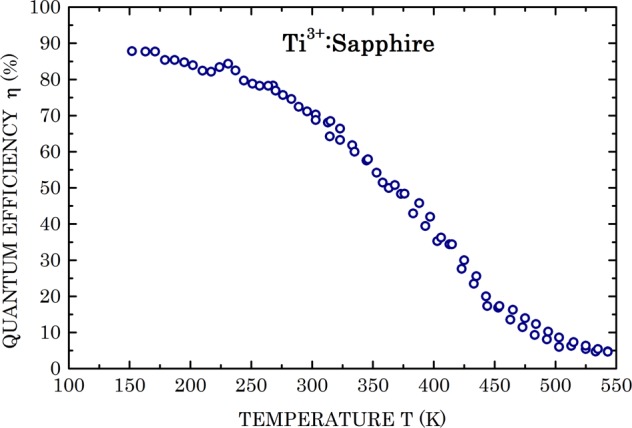
Fluorescence quantum efficiency of the Ti^3+^ ions in the sapphire matrix, normalized to 69.5% at 300 K, as function of absolute temperature. Data scatter about theoretical fits of the entire range (Figs. 4 and 5) contributes an error of ±0.02%.

In Fig. 3 the measured results of the fluorescence quantum efficiency as function of temperature are presented. At 150 K, the quantum efficiency is about 88%, there are no data for lower absolute temperatures; however, there appears a tendency for some moderate increased efficiency at lower temperatures. In view of the reducing slopes at the low temperature regions, an extrapolated efficiency of (88 ± 2)% in the T → 0 K limit is quite reasonable. That error figure includes the ±1% error of the normalization procedure of the entire curve to the single quantum efficiency datum provided in^13^. For temperatures exceeding 150 K, the quantum efficiency reduces quite steeply, reaching about 5% at 550 K.

Obviously, ${\tau }_{eff}^{-1}$ (*T*) = ${\tau }_{rad}^{-1}$ (*T*) + ${\tau }_{{nr}}^{-1}$ (*T*), where ${\tau }_{eff}^{-1}$ (*T*) is the measured fluorescence decay rate, ${\tau }_{rad}^{-1}$ (*T*) is the radiative transition rate and ${\tau }_{{nr}}^{-1}$ (*T*) is the non-radiative transition rate. Also, the fluorescence quantum efficiency is given by *η*(*T*) = *τ*_*eff*_(*T*)/*τ*_*rad*_(*T*). Thus

3 $${\tau }_{rad}(T)=\frac{{\tau }_{eff}(T)}{\eta (T)};\,{\tau }_{nr}(T)=\frac{{\tau }_{eff}(T)}{1-\eta (T)}.$$

**Figure 4:**
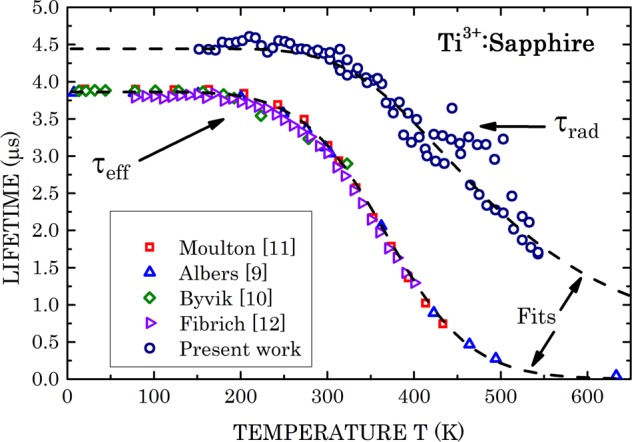
Comparison between literature data of the Ti^3+^:sapphire effective fluorescence decay time (*τ*_eff_) and the radiative lifetime (*τ*_rad_) measured in the present paper, both as functions of absolute temperature. Dashed lines are theoretical fits for *τ*_eff_ and *τ*_rad_ per present paper analysis (Eqs. (4) and (10)).

**Figure 5:**
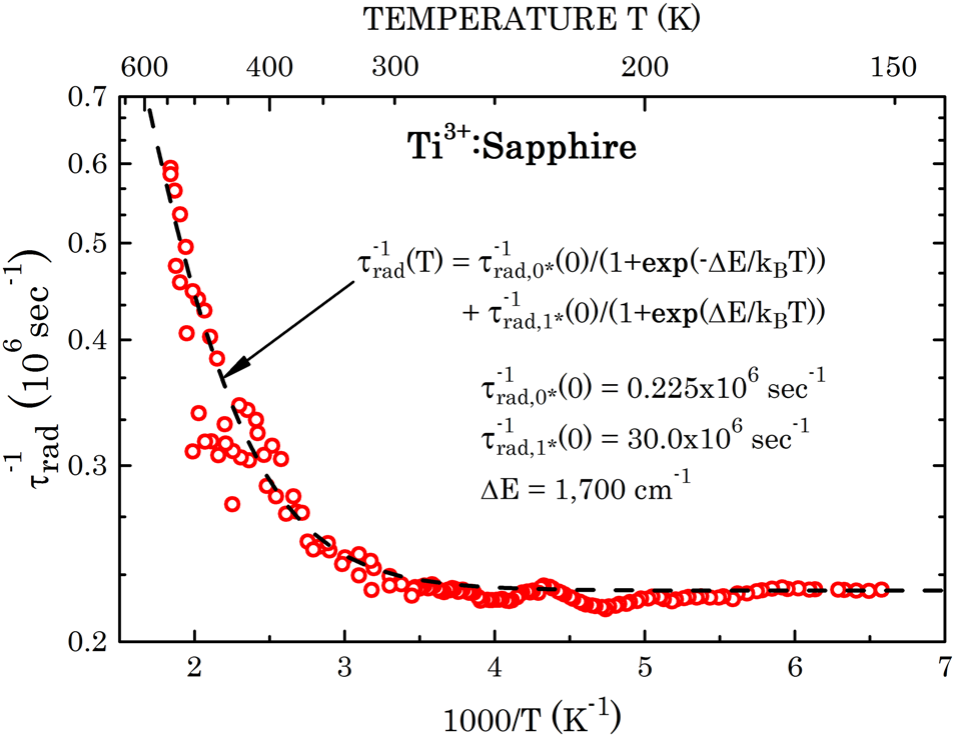
Semi-logarithmic plot of the fluorescence radiative decay rate *τ*_rad_^−1^(T) of Ti^3+^ ions in the sapphire matrix as function of 1000/T. Fit parameters per Eq. (4) are inset in the figure frame.

Using literature effective lifetime measurements performed by several authors^9–12^ allows direct calculations of the radiative fluorescence lifetime. The said literature effective lifetime data, and calculated radiative lifetime per Eq. (3), are plotted in Fig. 4. The radiative lifetime is about 15% longer than the effective lifetime in the low temperature region (150–200 K) and appears to shorten steeply with growing temperature. However, the radiative lifetime departure from the effective lifetime grows, becoming over 20 times longer than the effective lifetime at 550 K. For analysis of the radiative lifetime temperature dependence, in Fig. 5 we plotted the radiative transition rate ${\tau }_{rad}^{-1}$ as function of 1000/T on a semi-logarithmic scale. We interpreted the enhanced radiative rate as function of temperature by involvement of transitions from the top excited state of the upper multiplet (1^*^ state in Fig. 2) that is supposed to exhibit a higher vibrational overlap with the ground multiplet states. The mathematical expression reads

4 $$\begin{array}{rcl}{\tau }_{rad}^{-1}(T) & = & {\tau }_{rad,{0}^{\ast }}^{-1}(T)+{\tau }_{rad,{1}^{\ast }}^{-1}(T)\\  & = & {\tau }_{rad,{0}^{\ast }}^{-1}(0)[\frac{1}{1+\exp (-\Delta E/{k}_{B}T)}]+{\tau }_{rad,{1}^{\ast }}^{-1}(0)[\frac{1}{1+\exp (\Delta E/{k}_{B}T)}],\end{array}$$

where ${\tau }_{{rad,0}\ast }^{-1}$ (*T*) is the radiative transition rate from the upper multiplet bottom electronic state (marked 0^*^ in Fig. 2) to the ground multiplet states; ${\tau }_{rad,{1}^{\ast }}^{-1}$(*T*) is the radiative transition rate from the upper multiplet top electronic state (marked 1^*^ in Fig. 2) to the ground multiplet states; Δ*E* is the energy separation between the top and bottom upper multiplet states, and *k*_*B*_ is the Boltzmann constant. The temperature dependent factors in the square parentheses provide the fractional thermal occupation of the excited top and bottom upper multiplet states, respectively. Excellent fit is obtained for ${\tau }_{rad,{0}^{\ast }}^{-1}(0)$ = 0.225 × 10^6^ sec^−1^, ${\tau }_{rad,{1}^{\ast }}^{-1}(0)$ = 30.0 × 10^6^ sec^−1^, and Δ*E* = 1,700 cm^−1^. The latter newly obtained value for the 0^*^ ↔ 1^*^ separation has been correspondingly incorporated into the energy scheme (Fig. 2). Arrows indicating expected 1^*^ → 0, 1, 2 radiative transitions, however, are not included to avoid overcrowding. The fit for *τ*_*rad*_(*T*) thus obtained has been incorporated into Fig. 4, and is shown by a dashed line throughout the temperature range of measured data, as well as extrapolated down to 0 K and up to 650 K. Notably, *τ*_*rad*_(0) = (4.44 ± 0.04) *μ*s compared to *τ*_*eff*_(0) = 3.85 *μ*s.

There are notable conclusions related to the said fit. The energy separation between the two upper multiplet electronic states, 1,700 cm^−1^, is smaller than the same separation obtained by absorption spectral measurement (2,430 cm^−1^, see Fig. 2). That is obviously a result of the Stokes-shift between absorption and emission spectra; the entire emission states energy scheme is squeezed down to between 70% and 80% of the absorption states energy scheme.

The radiative decay transition rate ${\tau }_{rad,{1}^{\ast }}^{-1}(0)$ is about 135 times faster than ${\tau }_{rad,{0}^{\ast }}^{-1}(0)$! Its value is of the order of an ideal classical electronic oscillator. The latter decay rate is given by *γ* ≡ 8*π*^2^*e*^2^/3*m*_*e*_*cλ*^2^, where *e* is the elementary charge value, *m*_*e*_ is the electronic rest mass, *λ* is the decay transition wavelength, and *c* is the vacuum speed of light constant. Setting *λ* ≈ 680 nm, one obtains *γ* = 48 × 10^6^ cm^−1^. In other words, the transition oscillator strength from the top upper multiplet state to the ground multiplet states (*f* = 0.62) is quite close to a unity!

The apparent small value of ${\tau }_{rad,{0}^{\ast }}^{-1}(0)$ might be accounted for by considering two factors. Firstly, the integrated measured absorption cross-sections of the 0 → 1^*^ transition is 3.15 times more intense than the 0 → 0^*^ one (see Fig. 2). The former initial state is related to the ${\tau }_{rad,{1}^{\ast }}^{-1}(0)$ rate, and is expected to be larger by reciprocity considerations. The other reason must be related to a drastic difference in the Franck-Condon overlap integrals of the radiative transitions. Recall that the transition electric dipole moment expectation value is given by

5 $$\langle \mu \rangle =\langle {S}^{f}|{S}^{i}\rangle \langle {\psi }^{f}|{\mu }_{e}|{\psi }^{i}\rangle ,$$

where $\langle {\psi }^{f}|{\mu }_{e}|{\psi }^{i}\rangle$ is the electronic transition dipole moment between the initial electronic state *i* and the final electronic state *f*; and $\langle {S}^{f}|{S}^{i}\rangle$ is the Franck-Condon overlap integral (integral overlap between the wave functions of atomic vibrations belonging to the initial level *i* and those belonging to the final level *f*). The transition rate probability is proportional to the squared electric dipole moment expectation value. The unavoidable conclusion is that the Franck-Condon offset of the Stokes shifted 1^*^ state is considerably different than the offset of the 0^*^ state! That is obviously an issue worthwhile exploring theoretically. The above mentioned oscillator strengths *f* are in fact each resolved into three linear contributions according to their optical weights in the emission spectrum (Fig. 2). We assume that the same weights exist for the radiative fluorescence emission from the 1^*^ state. Resolution of *f* ’s and ${\tau }_{rad}^{-1}$ ’s are summarized accordingly in Table 1. This resolution is important for our next analysis of the non-radiative transitions.

**Table 1:** Details of oscillator strength and radiative transition rate between states of upper and lower multiplets.

final (*f*)	2		1		0	
initial (*i*)	*f*	${{\boldsymbol{\tau }}}_{{\boldsymbol{rad}}}^{-{\bf{1}}}$ (10^6^ *s*^−1^)	*f*	${{\boldsymbol{\tau }}}_{{\boldsymbol{rad}}}^{-{\bf{1}}}$ (10^6^ *s*^−1^)	*f*	${{\boldsymbol{\tau }}}_{{\boldsymbol{rad}}}^{-{\bf{1}}}$ (10^6^ *s*^−1^)
0^*^	0.258 × 0.013	0.258 × 0.225	0.51 × 0.013	0.51 × 0.225	0.232 × 0.013	0.232 × 0.225
1^*^	0.258 × 0.62	0.258 × 30.0	0.51 × 0.62	0.51 × 30.0	0.232 × 0.62	0.232 × 30.0

The ratios of 0^*^ to 2, 1, 0 lower multiplet states transitions are 0.258, 0.51 and 0.232, respectively (see Fig. 2). Same relations are assumed for the 1^*^ to 2, 1, 0 transitions. Oscillator strengths and radiative transitions rates are resolved accordingly.

The assignment of enhanced radiative transitions to a higher electronic state in the upper multimpl*et al*so means that additional fluorescence transitions set in upon increase of temperature. So far, high temperature fluorescence measurements were limited to below 400 K (Fig. 3 in^12^). Indeed, the fluorescence peak at 760 nm becomes relatively smaller with increasing temperature, while, especially for a *σ*-polarization, a peak located at 690 nm grows more prominent. The latter wavelength is consistent with a 1^*^ → 1 prospected transition (Fig. 2). That observation supports an interpretation of the 1^*^ state to be located 1,700 cm^−1^ above the 0^*^ state. It also supports the 1^*^ state role in the radiative transition participation and its dominance in the radiative lifetime shortening upon increase in temperature. Extension of fluorescence spectral measurements towards higher temperatures, such as 550 K, is highly desirable.

In the particular simple case of the non-radiative transition between two separate states, the non-radiative transition rate *τ*_*nr*_^−1^ is given by^15,16^

6 $${\tau }_{nr}^{-1}={\tau }_{rad}^{-1}\,{C}_{nr}\,{(1+\langle n(T)\rangle )}^{\upsilon };\,n(T)=\frac{1}{\exp (\hslash {\omega }_{co}/{k}_{B}T)-1},$$

where 〈*n*(*T*)〉 is the thermal average occupation number of $\hslash$*ω*_*co*_ energy phonons, $\hslash$*ω*_*co*_ being the cutoff vibrational energy in the crystal, (measured^25^ as 908 cm^−1^), *C*_*nr*_ is Burshtein’s multi-phonon non-radiative coefficient factor, which has been theoretically calculated^15^ as

7 $${C}_{nr}=\frac{\hslash c}{{e}^{2}}\,\frac{{N}_{c}-1}{{N}_{c}}\,\frac{2{\pi }^{2}}{{n}^{3}}\frac{[{\bar{M}}_{at}{c}^{2}]\hslash {\omega }_{co}}{{E}_{g}^{2}}B(\upsilon ){(\frac{\hslash {\omega }_{co}}{D})}^{\upsilon -1},$$

where $\hslash$ is the reduced Planck constant, *c* is the vacuum speed of light constant, *e* is the elementary charge value, *N*_*c*_ is the number of atoms occupying the primitive unit cell (correspondingly forming the ligand for each embedded ion), *n* is the ion host matrix refractive index, ${\bar{M}}_{at}$ is the average atomic mass in the unit cell, *ω*_*co*_ is the cut-off angular frequency of the matrix vibrations (phonons), *E*_*g*_ is the energy gap between two initial and final separated states, *D* is the characteristic dissociation energy of a Morse-type configuration potential^26^ (related to the primitive unit cell vibrating in free-space at *ω*_*co*_ angular frequency), *υ*, defined as *υ* ≡ *E*_*g*_/$\hslash$*ω*_*co*_ represents the number of $\hslash$*ω*_*co*_ phonons involved in the non-radiative transition. The 〈*n*(*T*)〉 quantity in Eq. (6) is the average thermal occupation number of $\hslash$*ω*_*co*_ phonons. The *B*(*υ*) function included in Eq. (7) above is a *υ*-dependent numerical factor; its presence in Eq. (7) stems from expansion of the assumed lattice configuration potential to a Taylor series, as well as from other model assumptions for the *υ*-order multi-phonon induced transitions^15^. It is given by

8 $$B(\upsilon )=\frac{{[{2}^{\upsilon }-1]}^{2}{\upsilon }^{\upsilon -2}}{{[\varGamma (\upsilon +1)]}^{2}}.$$

The ratio ($\hslash$*ω*_*co*_/*D*) ≡ $\hat{H}$ appearing in Eq. (7) may be interpreted as a figure-of-anharmonicity of the lattice vibrations; in a perfectly harmonic potential, the ($\hslash$*ω*_*co*_/*D*) ratio diminishes, and correspondingly, in that limit the non-radiative transition rate also diminishes (Eq. (7)). Obviously, it is always small compared to a unity.

In our present case one has to consider the involvement of non-radiative transitions from all upper multiplet states to all ground multiplet states; 0^*^ → 2, 1, 0 and 1^*^ → 2, 1, 0 (see Fig. 2). The total non-radiative transitions would thus be of the form

9 $${\tau }_{nr}^{-1}(T)=\mathop{\sum }\limits_{f=0}^{2}\,[{\tau }_{rad,{0}^{\ast }f}^{-1}(T){C}_{nr}^{({0}^{\ast }f)}\,(1+\langle n(T)\rangle {)}^{{\upsilon }_{{0}^{\ast }f}}+{\tau }_{rad,{1}^{\ast }f}^{-1}(T){C}_{nr}^{({1}^{\ast }f)}\,(1+\langle n(T)\rangle {)}^{{\upsilon }_{{1}^{\ast }f}}],$$

where *υ*_0*f_ belongs to 0^*^ → 2, 1, 0 transitions and *υ*_1*f_ belongs to 1^*^ → 2, 1, 0 transitions.

**Figure 6:**
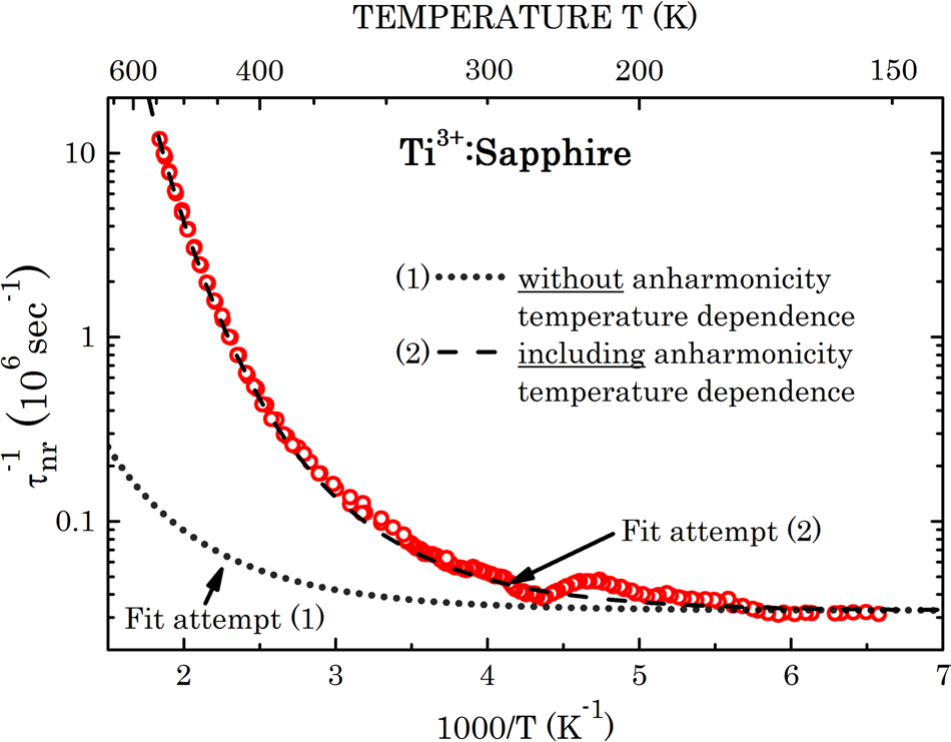
Semi-logarithmic plot of the non-radiative fluorescence decay rate *τ*_nr_^−1^(T) of Ti^3+^ ions in the sapphire matrix as a function of 1000/T. Dotted line is a fit based on our previous basic theory^16^, dashed line is a fit that further assumes a temperature dependence of the configuration potential anharmonicity. Fit parameters per Eq. (9) and Eq. (10) are summarized in Table 2.

A fit attempt of Eq. (9) to the experimental results is shown by a dotted line in Fig. 6. The following basic material parameters of Ti:sapphire were used: ${\bar{M}}_{at}$ = 20.3922 amu, *N*_*c*_ = 10, $\hslash$*ω*_*co*_ = 908 cm^−1^ ^25^, *n* = 1.761, and $\hat{H}$(0) assumed as 0.276 (a fit at T → 0 K; correspondingly *D*(0) = 3,285 cm^−1^). Other process parameters are listed in Table 2.

**Table 2:** Process parameters used for fitting Ti:sapphire *τ*_nr_^−1^(T) to Eqs. (9) and (10).

j^*^	*f*	${{\boldsymbol{E}}}_{{\boldsymbol{g}},{{\boldsymbol{j}}}^{\ast }{\boldsymbol{f}}}$(cm^−1^)	*υ*_j*f_	*B*(*υ*_j*f_)	${{\boldsymbol{\tau }}}_{{\boldsymbol{rad}},{{\boldsymbol{j}}}^{\ast }{\boldsymbol{f}}}^{-{\bf{1}}}$(0) (10^6^ s^−1^)	*C*_*nr*_^(j*f)^ (0)
1^*^	2	13,190	14.50	1.575	0.258 × 30.0	1.58 × 10^−2^
	1	14,600	16.10	0.665	0.51 × 30.0	7.42 × 10^−4^
	0	15,800	17.40	0.300	0.232 × 30.0	5.23 × 10^−5^
0^*^	2	11,490	12.65	3.495	0.258 × 0.225	5.05 × 10^−1^
	1	12,900	14.20	1.820	0.51 × 0.225	2.88 × 10^−2^
	0	14,100	15.50	0.935	0.232 × 0.225	2.26 × 10^−3^

Apparently, the attempted fit fails to exhibit a sufficiently steep rise of the experimental *τ*_*nr*_^−1^(*T*) with increased temperature (reduced reciprocal temperature). We attribute this enhanced temperature dependence to an increase with temperature of the figure-of-anharmonicity of the configuration potential $\hat{H}$ (see above). The attempted functional dependence is assumed as

10 $$\hat{H}(T)=\hat{H}(0)(1+\beta \,\exp (-\hslash {\omega }_{co}/{k}_{B}T)),$$

where $\hat{H}$(0) is the zero-temperature figure-of-anharmonicity, and *β* is an empiric fit parameter. Excellent fit is obtained for $\hat{H}$(0) = 0.276 and *β* = 5.2; see fit attempt (2) in Fig. 6.

Temperature enhancement of vibrational anharmonicity in crystals is a well known effect. It particularly expresses itself in causing the ferroelectric-to-paraelectric transition upon temperature increase of ferroelectric materials^27–29^. Equation (10) above provides an empiric account of the thermal anharmonicity enhancement in sapphire crystals. It is quite instructive to realize that such effect plays an important role in influencing of non-radiative fluorescence losses.

The appearance of the phonon vibrational cut-off energy ($\hslash$*ω*_*co*_) in the exponential factor addend in Eq. (10), identically for all contributing non-radiative transitions (0^*^ → 2, 1, 0 and 1^*^ → 2, 1, 0), provides a strong motivation to suggest that this parameter is practically temperature independent across the studied temperature range. In that case, the significant temperature dependence is attributed to the characteristic dissociation energy of the configuration potential parameter *D*. It would thus be approximately given by *D*(*T*) ≈ *D*(0)/(1 + *β* exp(−$\hslash$*ω*_*co*_/*k*_*B*_*T*)). It should still be valuable to measure the cutoff frequency dependence on temperature by, for example, neutron inelastic scattering, or by infrared surface reflectance.

## Summary and Conclusions

We have measured the fluorescence quantum efficiency in Ti^3+^:sapphire single crystals between 150 K and 550 K. A solid-angle fraction of fluorescence emission power was measured against an absorbed power in the sample under illumination at 543 nm. The fluorescence quantum efficiency was derived from the ratio between the estimated total fluorescence photon intensity and the absorbed photon intensity. We showed that the zero temperature radiative lifetime is (4.44 ± 0.04) *μs* compared to the fluorescence 3.85 *μs* lifetime. The fluorescence lifetime thermal shortening is interpreted to result from two parallel effects: radiative lifetime shortening and non-radiative transition rate enhancement. The first is due to enhanced thermal occupation of a Δ*E* = 1,700 cm^−1^ higher (top) electronic state of the upper manifold exhibiting a high transition oscillator strength of ***f*** = 0.62 compared to only 0.013 of its bottom electronic state. The non-radiative rate relates to multi phonon decay transitions stimulated by the lattice thermal phonon occupation. The latter effect analysis provides an estimate of the “dissociation-energy” of the zero-temperature configuration potential *D*(0) = 3,285 cm^−1^. An empiric expression for a related anharmonicity measure of the configuration potential is given as $\hat{H}$(*T*) = $\hat{H}$(0)(1 + *β* exp(−$\hslash$*ω*_*co*_/*k*_*B*_*T*)), where $\hat{H}$(0) = 0.276, *β* = 5.2, and $\hslash$*ω*_*co*_ = 908 cm^−1^.

The results support the usefulness and validity of a model that relates the non-radiative decay transitions of excited states to multi phonon transitions in the lattice vibrational field.

It also shows that a combined study of fluorescence decay time and corresponding fluorescence quantum yield, both as functions of temperature, allows the discrimination between radiative and non radiative decay rates. Moreover, it allows insight into the non-radiative multi phonon transitions. It could further serve as a probe of anharmonicity and its temperature dependence in dielectric crystals. Study of radiative and non-radiative transitions of embedded ions in a variety of dielectrics is highly desirable.

A number of issues related to Ti:sapphire proper and sapphire in general are worthwhile addressing specifically. One is a geometric nature assessment of the C_3_ site distortion by the Ti^3+^ when it replaces Al^3+^. A related topic would be a theoretical assessment of the contribution of Jahn-Teller’s effect to the splitting of the ground ^2^*E* state. A further issue for theoretical consideration is calculation of the Franck Condon overlap integrals between upper and ground multiplet states. Another one is an experimental assessment of the temperature dependence of lattice vibrational cut-off frequency *ω*_*co*_ (considered temperature independent in our present study). A measurement of the linear thermal expansion coefficient could validate the anharmonicity temperature dependence suggested by our experimental results. Finally, Ti:sapphire fluorescence measurement at temperatures higher than 400 K, especially for *σ*-polarization, could also assist in strengthening the model and conclusions of the present work.
